# In Memoriam—Edward Bayard, M. D.

**Published:** 1889-12

**Authors:** 


### IN MEMORIAM—EDWARD BAYARD, M. D.

Dr. Edward Bayard, who for nearly fifty years has been one of the leading homœopathic physicians of the world, passed from his labors Saturday, September 28th, 1889, at North Yarmouth, Me., at the age of 83 years, 6 months, 23 days.

The Doctor had been in excellent health till the 24th of November, 1888, when he sustained a serious injury from a fall while arising at night; this confined him to his house all winter. With the warm weather he improved, and in July left for North Yarmouth to spend the summer. The pure air and freedom from his extensive practice produced a favorable change, and arrangements had been made for his return, when a sudden change in the weather occurred, causing the development of an acute attack of bronchitis, which he was unable to withstand.

Dr. Bayard was born March 5th, 1806, at Wilmington, Del., and was the third son of the Hon. James A. Bayard, one of the United States Commissioners who negotiated the treaty of Ghent. His brothers were the late United States Senators, Richard and James Bayard, while he was uncle to ex-Secretary of State Thomas F. Bayard. He graduated at Union College, Schenectady, in 1825, and began the study of law in the office of Judge Daniel Cady, at Johnstown, N. Y., whose eldest daughter he afterward married.

While at Union College, he organized the students as a cadet corps, himself being in command. During this time there was a strong feeling of prejudice among the populace, which culminated in an uprising against the college. Dr. Knott, the President of the college, called upon young Bayard to lead the students against the mob, and it was only through his efforts that the college was preserved. This incident in his early career was but the indication of his strong will and determined character, which in after life distinguished him so greatly above his fellows.

He was admitted to the Bar as attorney-at-law in 1828, and as a solicitor to the Court of Chancery in 1835.

August 1st, 1835, he was made Lieutenant-Colonel of the Thirty-fourth Regiment of the New York State Militia.

After practicing law at Johnstown for a short time, he removed to Seneca Falls, where he entered into partnership with Judge Foot, and established a large and successful practice; but in the midst of his labors his health gave way, and he was so troubled with palpitation of the heart that he came to New York to consult Dr. Stevens, a noted specialist of that day, who told him that he must retire from all active employment, and lead a quiet and uneventful life, as he had a heart trouble which would eventually cause his death. At the earnest solicitation of Mrs. Bayard, he consulted Dr. Biegler, of Albany, who made a most careful examination of his condition, and asked him if he was a coffee drinker. Upon receiving an affirmative reply, the Doctor asserted that this was the cause of the trouble, and advising the discontinuance of coffee, giving him a few pellets of the homœopathic remedy on his tongue, with some powders, told him he would soon be well.

Returning to his home, he soon regained his former vigor; but the result of the treatment he had received impressed him so profoundly, that he took up the study and practice of Homœopathy. So successful was he in this, the demand upon his time became so great, that he abandoned the Law and thenceforward devoted himself to Medicine. He went to New York, entered the University Medical College, graduated in 1844, and located permanently in that city.

Dr. Bayard was one of the early members of the American Institute of Homœopathy, and one of the founders of the Homœopathic Medical Society of the County of New York.

April 5th, 1850, he was made corresponding member and Fellow of the Homœopathic Medical College of Pennsylvania, at Philadelphia, in consideration of his high medical attainments and skill. In 1851, the Western Homœopathic College, of

Cleveland, Ohio, conferred upon him the honorary degree of Doctor of Medicine. In 1856, he was elected a member of the Geographical and Statistical Society of New York. In 1881, he joined the International Hahnemannian Association, and was one of its most earnest friends and supporters. The New York Homœopathic Union, formed in 1888 for the purpose of studying the *Organon*, was in large measure due to his interest in the work; he was its first President, and gave it the name it bears.

Alas! our dear old friend is no more. His sufferings are over, and we must not grudge him the rest, which, after his long and useful life, he so richly deserved. He had many amiable qualities which will endear him to our memory as long as we live. His sagacity, his knowledge, his sincerity, his courage, made him in our homœopathic circle, the chevalier *sans peur et sans reproche*, who never failed to step in the place of danger to rescue and uphold the sacred cause to which he devoted his life. He can never be replaced.

He was a steadfast, unvarying friend, and we mourn his demise as deeply on account of our personal loss as on that of our common cause, of which he was just as sincere and unvarying a supporter. No doubt his legal training before he embraced the great vocation of a homœopathician was a great help to him in his practice, for *qui bene judicat, bene curat. Integritas judicii, fons aud caput bene medendi.* All those who knew him testify to his accuracy in prescribing, which made him so successful in his clinical capacity. There was in him that affability of manner, that humane amiability of temper, which won the heart of his patients at the first glance and procured for him their full confidence. Seldom a physician was so loved by his compeers and his patients as he. But he excelled not merely as a practical homœopathician; his range of vision went over the whole field of science, and this made his actions so much more valuable and important, because he drew from the full treasure of his well-stocked mind.

When the testimony as to the superior action of the high potencies over the low potencies and crude drugs came in with overwhelming force, the Doctor was one of the first to test them, and after finding the evidence true, he availed himself thenceforth of these new means of healing the sick.

We need not allude to his prompt action when Homœopathy was in danger of being plowed under by the misguided efforts of a younger generation who comprehended it not, and when his legal ability stood him in good stead; nor need we remind the profession of the provings of Rhus radicans, which rendered him a martyr to a prosopalgia, torturing him for over thirty years, from time to time, and embittering his life. Certain it is that the star which rose with him on the homœopathic firmament was one of the first magnitude, and will shine as a comfort and encouragement for the votaries of the cause of homœopathics when the host of adversaries try to extinguish its light which still illuminates the darkness of medicine.

Deep as was the Doctor's knowledge of his art, his skill in the administration of the remedy, yet he had the greater knowledge and comprehension of the fundamental elements embraced in its philosophy. Few there are among his contemporaries who took so deep an interest in that branch of the profession, and fewer still those who studied it so deeply ; his learning was profound, his understanding comprehensive, his elucidation clear, and those who listened to him knew that here was one to whom Hahnemann was an open book.

The Rev. Dr. Dyer, who conducted the funeral services, in paying a beautiful tribute to his friend and physician, said: "That although his family had been distinguished for three generations, no member had added greater lustre to the name than he whom we were assembled to honor." He was a devout Christian, whose faith was shown in every act of his life. His tender treatment of his poorest patients endeared him to all who knew his boundless charity and faithful services given the multitude "without money and without price." The members of his family, many now in the sunset of life, in discussing his characteristics, alike testify to his uniform kindness and self-control ; none could recall a harsh word or act toward brother or sister in their long and intimate domestic life. A sweet charity and divine patience pervaded alike his social and professional life.

When mid singing birds and gentle breezes and the glad sunshine, nature takes our loved ones to her bosom, to be seen no more, the parting is hard to bear; but a pouring rain with heavy thunder intensified our grief as we left our noble brother's form alone in its last resting place.

No amount of philosophy can enable us to wholly divest our minds of the thought that the dead feel as we do the solitude of the grave.

